# Cumulative incidence and risk factors for radiation induced leukoencephalopathy in high grade glioma long term survivors

**DOI:** 10.1038/s41598-021-89216-1

**Published:** 2021-05-13

**Authors:** Robert Terziev, Dimitri Psimaras, Yannick Marie, Loic Feuvret, Giulia Berzero, Julian Jacob, Caroline Dehais, Flavie Bompaire, Wolf Mueller, Ben Kinnersley, Jean-Yves Delattre, Ahmed Idbaih, Khe Hoang-Xuan, Marc Sanson, Damien Ricard

**Affiliations:** 1 Service de Neurologie 2-Mazarin, Groupe Hospitalier Pitié-Salpêtrière, AP-HP, 75013 Paris, France; 2 Inserm, CNRS, UMR S 1127, Institut du Cerveau, ICM, équipe génétique et développement des tumeurs cérébrales, labellisée Ligue Nationale contre le Cancer, Sorbonne Université, 75013 Paris, France; 3 Service de Radiothérapie, Groupe Hospitalier Pitié-Salpêtrière, AP-HP, 75013 Paris, France; 4 Service de Santé des Armées, Hôpital d’Instruction des Armées Percy, Service de Neurologie, 101 boulevard Henri Barbusse, 92140 Clamart, France; 5 Université Paris-Saclay, Ecole Normale Supérieure Paris-Saclay, Service de Santé des Armées, CNRS, Université de Paris, UMR 9010 Centre Borelli, Paris, France; 6 Ecole du Val-de-Grâce, Service de Santé des Armées, Paris, France; 7 Department of Neuro-Pathology, University of Leipzig, 04103 Leipzig, Germany; 8 The Institute of Cancer Research, London, England SW7 3RP UK; 9 Site de Recherche Intégrée sur le Cancer (SiRIC), “CURAMUS”, 75013 Paris, France; 10 OncoNeuroTox Group: Center for Patients with Neurological Complications of Oncologic Treatments, Hôpitaux Universitaires Pitié-Salpêtrière-Charles Foix et Hôpital d’Instruction des Armées Percy, Paris, France; 11 Present Address: Department of Neurology and Brain Tumor Center, University Hospital Zurich and University of Zurich, Zurich, Switzerland

**Keywords:** Molecular medicine, Risk factors, CNS cancer, Oncology, Cancer therapy, Radiotherapy, CNS cancer, Neurotoxicity syndromes, White matter disease

## Abstract

The incidence and risk factors associated with radiation-induced leukoencephalopathy (RIL) in long-term survivors of high-grade glioma (HGG) are still poorly investigated. We performed a retrospective research in our institutional database for patients with supratentorial HGG treated with focal radiotherapy, having a progression-free overall survival > 30 months and available germline DNA. We reviewed MRI scans for signs of leukoencephalopathy on T2/FLAIR sequences, and medical records for information on cerebrovascular risk factors and neurological symptoms. We investigated a panel of candidate single nucleotide polymorphisms (SNPs) to assess genetic risk. Eighty-one HGG patients (18 grade IV and 63 grade III, 50M/31F) were included in the study. The median age at the time of radiotherapy was 48 years old (range 18–69). The median follow-up after the completion of radiotherapy was 79 months. A total of 44 patients (44/81, 54.3%) developed RIL during follow-up. Twenty-nine of the 44 patients developed consistent symptoms such as subcortical dementia (n = 28), gait disturbances (n = 12), and urinary incontinence (n = 9). The cumulative incidence of RIL was 21% at 12 months, 42% at 36 months, and 48% at 60 months. Age > 60 years, smoking, and the germline SNP rs2120825 (PPARg locus) were associated with an increased risk of RIL. Our study identified potential risk factors for the development of RIL (age, smoking, and the germline SNP rs2120825) and established the rationale for testing PPARg agonists in the prevention and management of late-delayed radiation-induced neurotoxicity.

## Introduction

The treatment of high-grade (i.e., World Health Organization—WHO—grade III and grade IV) gliomas (HGG) includes surgery, radiotherapy (RT), and chemotherapy, which are associated with a median overall survival of 72 months in grade III and 14 months in grade IV gliomas^1^. Although necessary for tumor control in HGG, radiotherapy is burdened by delayed toxicities that impair the quality of life of survivors. Radiation-induced leukoencephalopathy (RIL)^2^ is the most common correlate of radiation toxicity and can be associated with a variety of neurological symptoms, including cognitive deficits, gait disturbances, and urinary incontinence, all reflecting an impairment of subcortical networks^3–5^. Albeit more frequent and devastating in patients receiving whole-brain irradiation, RIL is also observed following focal RT, with similar clinical consequences^3^. The recent increase in the survival rates of patients with HGG has raised some concerns on the need to adopt adequate measure for the prevention and management of late-delayed toxicities in this population, which are primarily related to brain irradiation. To date, there are few published data on the incidence of radiation-induced leukoencephalopathy (RIL) in long surviving HGG patients and, although some individual susceptibility factors have been suggested (e.g., age, vascular risk factors, genetic predisposition)^2,6^, none has been convincingly demonstrated.

In this retrospective study, we investigated the cumulative incidence of RIL in a cohort of long surviving HGG patients, with the aim of identifying individual risk factors for the development of this condition.

## Methods

The data used in the study were obtained from the database of the Neuro-oncology Department at Hospital Pitié-Salpêtrière (Paris, France, OncoNeuroTek). The study was approved by the Comité de Protection des Personnes Paris Ile de France VI. The authors received the authorization for analysis from the ethical committee (CPP Ile de France VI, ref A39II) and the French Ministry for research (AC 2013-1962). Written informed consent for study participation was obtained. Inclusion criteria were: (1) age ≥ 18 years, (2) supratentorial WHO grade III or IV glioma treated with focal radiotherapy, (3) at least 30 months of progression-free survival following the first day of radiotherapy, (4) written informed consent, (5) availability of germ-line DNA for SNPs genotyping. Patients were excluded if their clinical record lacked sufficient data on treatment modalities or clinical evolution and if brain MRI was not performed within 3 months before RT (considered the baseline for the purposes of this study) and then regularly at 6-month intervals up until the date of last follow-up. RANO criteria for high grade gliomas^7^ were used to verify that none of patients included in the study met the criteria for tumor progression. Patients developing tumor progression or other condition that could account for novel neurological symptoms (e.g., Normal Pressure Hydrocephalus (NPH), vascular dementia) were excluded from the study. Lumbar puncture was performed in patients with clinical and/or MRI suspicion of Normal Pressure Hydrocephalus (NPH) (assessed by sensible gait speed improvement after at least one CSF subtraction).

All MRI scans performed during follow-up were reviewed independently by two investigators (RT, DR). In case of discordant assessment, the investigators discussed the cases together and reached a consensus. Each MRI scan performed during follow-up was analyzed, comparing it to previous scans in order to ascertain accurately the first appearance of RIL and its evolution over time. RIL was defined by the following items: (1) focal or confluent, T2w-hyperintense white matter changes, in the area surrounding the surgical cavity and residual tumor, extending to at least one other lobe or bilaterally to the periventricular white matter; (2) cortical and subcortical atrophy adjacent to white matter changes; (3) score ≥ 10 at the modified Scheltens rating scale (Table 1)^8^ for white matter changes. The diagnosis of RIL was made, if all three criteria were met at one point of the follow up.

**Table 1:** Rating of FLAIR hyperintensities according to a modified Scheltens rating scale.

**Modified Scheltens rating scale**		
Periventricular hyperintensities (/6)		
Occipital caps	0/1/2	1: right or left hemisphere confluent lesion 2: bilateral confluent lesion
Frontal caps	0/1/2	
Lateral ventricle bands	0/1/2	
White matter hyperintensities (/10)		
Frontal	0/1/2	1: right or left hemisphere confluent lesion 2: bilateral confluent lesion
Occipital	0/1/2	
Temporal	0/1/2	
Insular	0/1/2	
Parietal	0/1/2	
Basal ganglia hyperintensities (/10)		
Caudate nucleus	0/1/2	1: right or left hemisphere confluent lesion 2: bilateral confluent lesion
Putamen	0/1/2	
Globus pallidus	0/1/2	
Thalamus	0/1/2	
Internal capsule	0/1/2	
Infratentorial foci of hyperintensities (/4)		
Cerebellum	0/1/2	1: right or left hemisphere confluent lesion 2: bilateral confluent lesion
Midbrain	0/1/2	

Medical records were consulted to obtain information on the first appearance and the evolution of symptoms of RIL, such as cognitive deficits, gait difficulties and urinary incontinence^3^, and on the presence of vascular risk factors, such as arterial hypertension, hypercholesterolemia, diabetes and smoking. All patients underwent a standardized neurological assessment: physical and cognitive complaint recording, visual assessment of gait, standing, segmental force and sensibility testing, language evaluation by object denomination and episodic memory test by 3 words learning. At the end of each clinical examination, the presence of neurological syndromes e.g. hemiparesis, cerebellar syndrome or extrapyramidal syndrome was recorded in the medical file. We took cognitive, gait or urinary deficits into account if they were mentioned in the medical files at 3 consecutive examinations in a 12 months delay and recorded the date of appearance of these deficits at the first mention in the medical files.

Single nucleotide polymorphisms (SNPs) genotyping was performed using Illumina Infinium HD Human610-Quad, as previously reported^9^. SNPs were selected searching the literature for three categories of candidate genes: (1) those associated with radiation-induced toxicity in organs other than the brain, (2) those involved in oligodendrocyte growth, and (3) those involved in reactive oxygen species elimination. This allowed to identify a list of 212 SNPs on 49 different genes^10–15^ that could be of interest in the setting of RIL (supplementary data). Statistical analysis was performed using R v3.4.2 (2017-09-28). A competing risk of death analysis was used to investigate the time-related RIL rate^16^. The potential risk factors assessed included SNPs, age, gender, smoking status, arterial hypertension, diabetes, and hypercholesterolemia. Multivariate analysis was performed using the Cox model as well as the Competing Risk Regression (CRR) model. The threshold for statistical significance was set at *p* value < 0.05. To avoid false positive findings due to multiple-testing, we controlled for all significant results using the false discovery rate (FDR)^17^ and considered it significant for q-values < 0.1. All Methods were performed in accordance with the relevant guidelines and regulations as mentioned in the policies of Nature Research journals.

## Results

Of 1391 patients with HGG included in our institutional database, 81 (50M/31F; sex ratio = 1.6) met inclusion/exclusion criteria (Table 2). As patients were long-term survivors, histopathologic diagnosis had been made before the 2016 WHO classification. Based on diagnoses at the time, the study included 44 anaplastic oligodendrogliomas (44/81, 54.3%), 14 anaplastic oligoastrocytomas (14/81, 17.3%), 5 anaplastic astrocytomas (5/81, 6.2%) and 18 glioblastomas (18/81, 22.2%). Genetic analysis for tumor *IDH1* mutational status and 1p/19q status was available for 57 patients: 20 patients were IDH1wt (35.1%), 19 were IDH1-mutant but had no 1p/19q codeletion, (33.3%), and 18 were IDH1-mutant and 1p/19q codeleted (31.6%).

**Table 2:** Patient characteristics.

Total number of patients	81
Sex ratio (f:m)	31:50
**Tumor subgroups**	
Anaplastic astrocytomas (AIII)	5
Anaplastic oligodendrogliomas (OIII)	44
Anaplastic mixed gliomas (mixed III)	14
Glioblastomas (GBM)	18
**Tumor location**	
Frontal	40 (49.4%)
Fronto-parietal	1 (1.2%)
Fronto-temporal	4 (5.0%)
Temporal	16 (19.8%)
Temporo-parietal	2 (2.5%)
Parietal	13 (16.0%)
Parieto-occipital	1 (1.2%)
Occipital	3 (3.7%)
Diencephalic	1 (1.2%)
Antiepileptic treatment, Y/N	60/21
Median age at the time of RT (range)	48 years (18;69)
**Median follow-up time after RT (range)**	78.8 mo. (22.3–285.8 m)
RT only	28 (34.6%)
Concomitantt RT	17 (21.0%)
CT following RT	36 (44.4%)
Smokers (Y/N/n.a.)	12/26/43
Patients with arterial hypertension (Y/N/n.a)	11/57/13
Patients with diabetes (Y/N/n.a)	5/63/13
Patients with hypercholesterolemia (Y/N/n.a)	10/58/13
**Number of patients with leukoencephalopathy (LCP)**	44
Median time to LCP (range)	14.9 m (1.2–180.6 m)
Median follow-up time after LCP onset	59.5 m (16.2–183.1 m)
**Number of patients with clinical symptoms**	32
Cognitive impairment	28
Gait disturbance	12
Urinary incontinence	9
Median time to symptoms after RT (range)	39.4 m (4.7–146.1 m)

Tumor locations were as follows: frontal (40/81, 49.4%), fronto-parietal (1/81, 1.2%), fronto-temporal (4/81, 5.0%), temporal (16/81, 19.8%), temporo-parietal (2/81, 2.5%), parietal (13/81, 16.0%), parieto-occipital (1/81, 1.2%), occipital (3/81, 3.7%), diencephalic (1/81, 1.2%).

All patients received focal brain RT, with total doses between 55–62 Gy by 1.8–2.0 Gy fractions. Radiotherapy was administered between 1991 and 2008, using a 2D (before 2005) or a 3D delivery technique (from 2005 onwards). The median age at the time of RT was 48 years (range 18–69 years). Twenty-eight patients (28/81, 35%) received RT alone, while the remaining patients received concomitant (17/81, 21%) or adjuvant (36/81, 44%) chemotherapy with temozolomide or nitrosoureas.

The median follow-up interval after the completion of radiotherapy was 79 months (30–285 months). A total of 44 patients (54.3%, 25 M/19F) developed evidence of RIL. The median delay between RT completion and the first appearance of RIL was 14.9 months (range 1.2–180.6).

**Figure 1:**
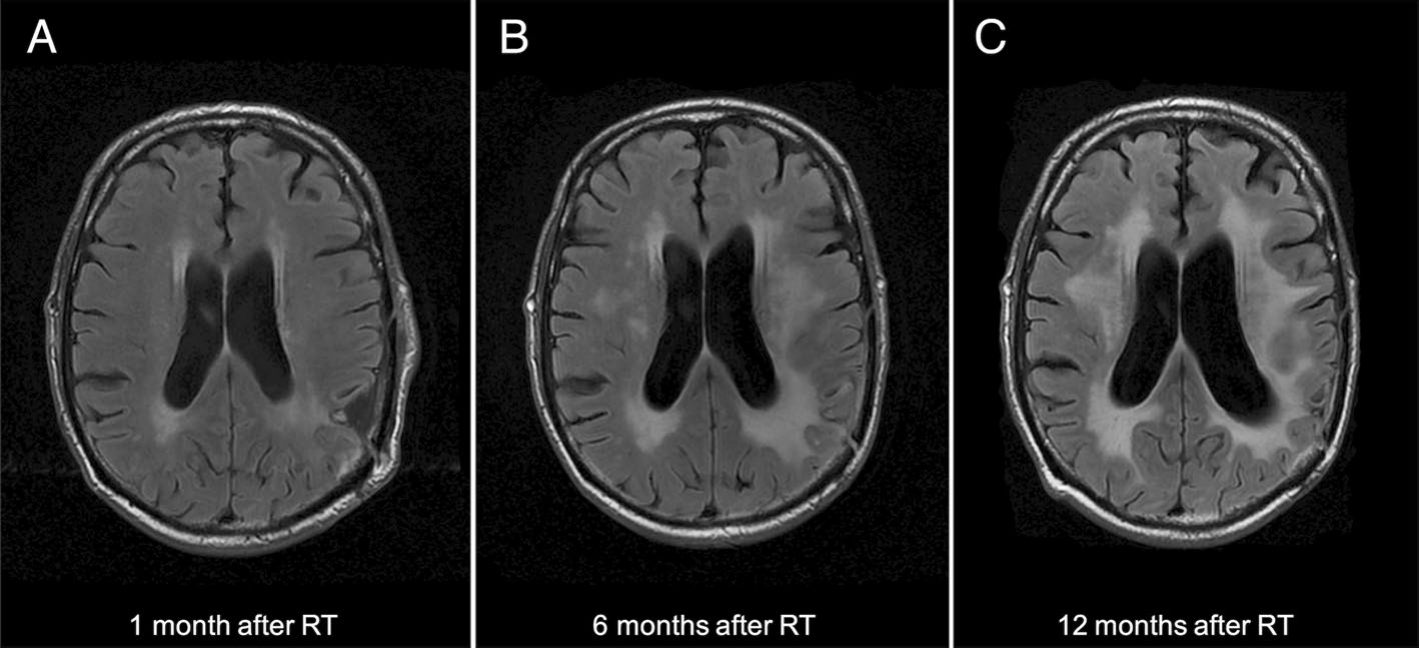
MRI findings of radiation-induced neurotoxicity presenting as rapidly progressing white matter hyperintensities associated with cortical and subcortical atrophy. The images show MRI findings at 1 month (**A**), 6 months (**B**) and 12 months (**C**) after RT. The patient received 60 Gy in 30 fractions with concomitant TMZ for a left parietal glioblastoma. After 1 year of follow up, memory deficits were present (MOCA 13/30) as were gait difficulties and urinary incontinence. The patients had the rs2120825 risk allele T (TG).

The MRI alterations corresponding to RIL first appeared in the peritumoral area then slowly extended to the ipsi- and contra-lateral white matter becoming confluent within few months for the 44 patients who developed RIL (Fig. 1). Among the 44 patients who developed RIL during the follow up, 29 (two thirds, 65.9%) developed consistent neurological symptoms. None of the patients considered to have developed RIL (according to our diagnostic criteria) displayed signs of mesencephalic microangiopathy, that may have indicated a microangiopathy of other origin.

Symptoms appeared with a median of 33 months (range 4.7–146.1) after the completion of RT, later than the first appearance of RIL on MRI. Cognitive deficits were the first symptom to appear present in all patients developing clinical manifestations of RIL, followed by gait disturbances and/or urinary dysfunction (median delay: 33 months vs. 44 months vs. 66 months) (Table 3). Initial cognitive complaints included decreased attention span, slowness, and memory deficits. Cognitive status deteriorated over time in 29/44 (65%) of patients with RIL on MRI, eventually fulfilling the criteria for overt dementia in 19 patients. A stereotypic gait disturbance characterized by small shuffling steps, a widened base, difficulties when turning, and postural instability developed in twelve of the 44 patients (12/44, 27.3%). Gait impairment progressed over months to years, causing patients to eventually become bedridden or wheelchair bound for 5 of them (5/44, 11%). Urinary incontinence was observed in the advanced phase in 9 patients and mimicked a frontal-type autonomic dysfunction (9/44, 20.5%).

**Table 3:** Number of patients who developed symptoms after 12 months, 36 months, and 60 months following radiotherapy.

Symptom	12 months	36 months	60 months
Overall	6/81 (7%)	15/75 (20%)	25/56 (45%)
Cognitive	5/81 (6%)	15/75 (20%)	22/56 (39%)
Gait disturbance	2/81 (2%)	2/75 (3%)	8/56 (14%)
Urinary incontinence	0/81 (0%)	1/75 (1%)	4/56 (7%)

**Figure 2:**
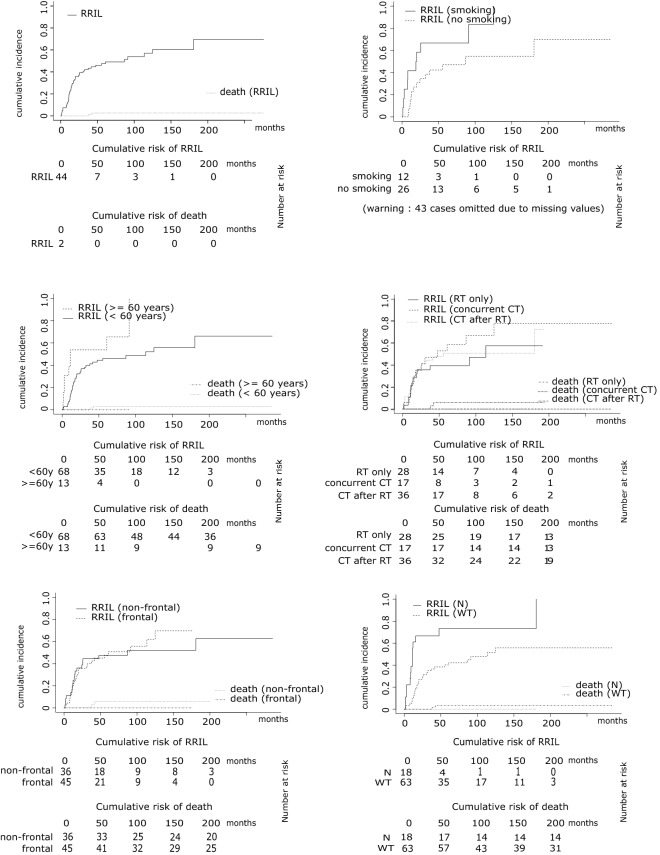
(**a)** Cumulative incidence of radiographical radiation-induced leukoencephalopathy (RRIL); FLAIR-hypersignal, enlargement of lateral ventricles or cortical atrophy corresponding to a modified Scheltens-score over 10; (**b)** cumulative incidence of RRIL in patients with a history of smoking and in non-smoking patients, n = 38; (**c**) cumulative incidence of RRIL in patients over age 60 (≥ 60 years) and patients under age 60 (< 60 years) at RT time; (**d**) cumulative incidence of RRIL in patients who received different treatments: RT only, RT with concomitant chemotherapy (NU or TMZ), or RT with chemotherapy following RT (NU or TMZ); (**e**) cumulative incidence of RRIL for frontal tumors and non-frontal tumors; (**f**) cumulative incidence of RRIL for PPARg variants (rs2120825): *N* non-wild-type (GG, TG), *WT* wild-type (TT).

The cumulative incidence of RIL was 21% at 12 months, 42% at 36 months, and 48% at 60 months (Fig. 2a), being higher in smoking than in non-smoking patients (at 12 months: 42% vs. 15%; at 36 months: 67% vs. 38%; at 60 months: 67% vs. 47%; *p* = 0.02; Fig. 2b). None of the other vascular risk factors considered in this study (i.e., arterial hypertension, *p* = 0.56; diabetes, *p* = 0.86; hypercholesterolemia, *p* = 0.81) were associated with a higher cumulative incidence of RIL (not shown).

Patients ≥ 60 years-old at the time of RT had an increased cumulative incidence of RIL compared to younger patients (at 12 months: 54% vs. 15%; at 36 months 54% vs. 40%; at 60 months: 54% vs. 46%; *p* = 0.032; Fig. 2c).

No significant differences in the cumulative incidence of RIL were observed based on the type of therapeutic regimen (i.e., RT alone, RT with concomitant chemotherapy, RT followed by adjuvant chemotherapy; Fig. 2d), although the curves start diverging after 2 years, suggesting that RIL may develop more often in patients who received additional (concomitant or adjuvant) chemotherapy.

Tumor location (frontal vs. non-frontal) had no impact on the incidence of RRIL (*p* = 0.72; Fig. 2e) or on the development of symptoms of RIL (*p* = 0.78).

We then assessed whether genetic variants in the 212 SNPs could affect the cumulative incidence of RIL. Among all SNPs included in the genetic analyses, only rs2120825 was associated with RIL. This SNP is located in the intron sequence of the Peroxisome-Proliferator-Activated-Receptor-Gamma (PPARg) gene. Patients with the T allele (TT/TG) had a higher cumulative incidence of RIL compared to patients without T allele (GG) (at 12 months: 61% vs. 10%; at 36 months: 67% vs. 35%; at 60 months: 73% vs. 40%; *p* = 0.0004, q-value 0.087, Fig. 2f). The frequency of the variant T allele was 12% in our population, the same than reported on the large European population of the DioGen (Larsen 2016, PMID: 26865646).

A first multivariate analysis using the Cox and the CRR model examined all suspected risk factors (sex, age, diabetes, hypercholesterolemia, hypertension, PPARg locus rs2120825) with only age and rs2120825 status being significant. Smoking was not included in the multivariate model because of missing data. A second analysis tested only the significant risk factors against each other. In all analyses, rs2120825 status was the only significant risk factor for RIL (Cox 1 *p* = 0.0097, HR = 2.8; Cox 2 *p* = 0.0055, HR = 2.6; CRR 1 *p* = 0.013, HR = 2.7; CRR 2 *p* = 0.005, HR = 2.6).

## Discussion

This is the largest study to date investigating the cumulative incidence and the risk factors associated with the development of RIL in HGG patients. We identified age, smoking and the germline SNP rs2120825 on the PPARg locus as risk factors for late radiation-induced toxicity. This information fills the gaps of previous reports^18–21^ and bears important repercussions in clinical practice, as some of these factors may be modified by lifestyle changes (e.g., smoking) or even by medication (e.g., PPARg agonists). The main limitations were related to the retrospective nature of our study, and included missing radiotherapy plans, to correlate the risk of RIL with radiation volume dose, and tumor location. The lack of systematic neuropsychological assessment may have led to an overestimation of asymptomatic RIL patients (one third in our study). Therefor we did not have sufficient data to properly analyze symptomatic patients and correlate to RIL features. But our clinical experience shows, that RIL precedes the symptoms and that all patients with RIL will develop symptoms eventually, if surviving and being followed long enough.

Only half of the patients in our series developed RIL with a high variability in the delay from the completion of RT (median 14 months) suggesting that there are individual factors affecting brain sensitivity to radiation. The extension and intensity of white matter changes was progressive over time until involving extensively the ipsi- and the contra-lateral hemisphere. Accompanying neurological symptoms appeared several months after the initial detection of RIL, according to a stereotyped sequence that starts with cognitive dysfunction followed by gait disturbances and urinary incontinence. The resulting clinical triad resembles that of other diseases that disrupt the subcortical circuits connecting the frontal cortex to basal ganglia^22,23^, such as Binswanger’s disease^24^, cerebral autosomal dominant arteriopathy with subcortical infarct and leukoencephalopathy (CADASIL) and normal pressure hydrocephalus^25^. Similarly to MRI abnormalities, neurological symptoms were in all cases progressive and resulted in a moderate to severe impairment in daily life activities. Not all patients with MRI evidence of RIL had accompanying symptoms, although minor complaints could have been under-reported in clinical records. Similarly, we did not find any correlation between the severity of RIL, measured on the modified Scheltens score, and the presence or severity of accompanying symptoms. Indeed the modified Scheltens scale is not specific for RIL and might not faithfully reflect clinical severity, as symptoms are not strictly associated to the extension and relevance of T2/FLAIR abnormalities but rather depend on brain atrophy or other white matter microstructure alterations not accessible to a standard MRI examination^26^.

We observed that age > 60 years at the time of RT was a significant risk factor for the development of RIL. This finding is consistent with a previous study on primary central nervous system lymphomas^4^.

Little is known about the cellular and pathophysiologic mechanisms^27^, but involvement of vascular damage^28^ or damage to oligodendrocytes or their precursor cells^28–30^ has been suggested.

Several vascular risk factors have been associated with the risk of radiation-induced central nervous system damage^2^. In our series, smoking was the only vascular risk factor associated with the development of RIL, while arterial hypertension, diabetes and hypercholesterolemia showed no significant association, but our study may be underpowered, given the limited amount of data available on cardio-vascular risk factors.

We identified one germline SNP (rs2120825), located at the PPARg locus associated with an increased risk of RIL. PPARg is a ligand-activated transcription factor belonging to the nuclear receptor superfamily. PPARg is highly expressed in vascular endothelial cells, and protect them from apoptosis^31^, atherogenesis^32^ and oxidative stress. PPARg is also implicated in the response of vascular smooth muscle cells to arterial hypertension and in the prevention of vascular wall deformations (e.g., aneurysms)^33^. In addition to its protective role in vasculature, PPARg modulates macrophage polarization and regulates the inflammatory responses of monocytes and microglial cells by inhibiting the liberation of nitric oxide synthase, matrix metallopeptidase-9, and several cytokines.

Finally, PPARg activation directly promotes oligodendroglial differentiation, enhances oligodendroglial survival, promotes myelin gene expression^34–37^, suppresses the inflammatory responses and reactive oxygen/nitrogen species and increases cellular anti-oxidants^34^ in oligodendrocytes.

All these considerations highlight the important role played by PPARg in endothelial cells, macrophages, microglia and oligodendrocytes, in the maintenance of cell trophism and survival, in the downregulation of inflammatory signals and in the elimination of reactive oxygen species. The observations that the damage induced by radiation highly depends on these mechanisms^2^ provided the rationale for testing PPARg agonist in the prevention and treatment of radiation-induced damage. The administration of PPARg agonists prevent radiation-induced toxicity and cognitive impairment in animal models^38–43^. The PPARg agonist pioglitazone, normally used as antidiabetic medication, has recently been experimented as neuroprotective agent in patients receiving brain irradiation, with good results in terms of tolerance^44^, paving the way to the experimentation of other drugs with PPARg agonist properties such as sartans^45^.

No other SNP, among the 212 tested, was found to be associated with an increased risk of RIL, including genetic polymorphisms, such as in *ApoE,* which are known affect the risk of developing Alzheimer’s disease and the risk of cognitive dysfunction in patients with brain tumors^5^.

## Conclusion

With the extended life expectancy that are experiencing HGG patients, the treatment of late-delayed toxicities will become an imperative. Fourteen months after RT (i.e., the median OS of glioblastoma patients^1^), 25% of our patients already showed signs of RIL. This observation provides a strong argument for further research on the individual risk factors for RIL. Firstly, this information will help clinicians to identify patients at high risk of developing RIL, adapting treatment schemes in patients with long-term life expectancy (e.g. 1p/19q codeleted gliomas). Secondly, the delay in the development of symptoms provides a therapeutic window of opportunity for correcting modifiable risk factors (e.g., smoking) and for administering neuro-protective agents that decrease vascular damage and oxidative stress in patients at higher risk. Our results, supported by available evidence in vitro and in vivo, provide further rationale for testing PPARg agonists in a prospective trial as neuroprotective agents.

Our study also points out the lack of cognitive tools routinely used to screen for the cognitive condition of high-grade glioma patients in order to validate the link between MRI RIL features and patients’ cognition. This need has previously been pointed out^46^. Recent randomised trials include long-term neurocognitive outcomes (for example EORTC study 22033–26033^47^; CATNON 2017^48^). Ongoing observational studies include NCT00457210^49^, NCT02544178^50^ and NCT03055364^51^. Such observational studies are aimed at clarifying the rate of late neurocognitive deficits in high-grade glioma patients and validating cognitive evaluation in these patients during their care process.

## Supplementary information

Supplementary Information 1.

Supplementary Information 2.

## Data Availability

Robert Terziev and Damien Ricard had full access to all the data in the study and take responsibility for the integrity and the accuracy of the data analysis.
