# Supplementary material for: Cumulative incidence and risk factors for radiation induced leukoencephalopathy in high grade glioma long term survivors
Source: Sci Rep. 2021 May 13;11:10176. doi: 10.1038/s41598-021-89216-1 (PMC8119685; doi:10.1038/s41598-021-89216-1)
Supplement: Supplementary file 1 — Supplementary Information 1. [file 41598_2021_89216_MOESM1_ESM.docx]

**Supplemetary Data**

**Cumulative incidence and risk factors for radiation-induced leukoencephalopathy in high-grade glioma long-term survivors**

Robert Terziev MD, Dimitri Psimaras MD, Yannick Marie PhD, Loic Feuvret MD, Giulia Berzero MD, Julian Jacob MD, Caroline Dehais MD, Flavie Bompaire MD, Wolf Mueller MD PhD, Ben Kinnersley PhD, Jean-Yves Delattre MD PhD, Ahmed Idbaih MD PhD, Khe Hoang-Xuan MD PhD, Marc Sanson MD PhD, *Damien Ricard MD PhD

**Genotyping**

DNA was extracted from samples using conventional methodologies and quantified using PicoGreen (Invitrogen, Carlsbad, USA). A genome-wide scan of tag SNPs was conducted using the Illumina Infinium HD Human610-Quad BeadChips according to the manufacturer's protocols (Illumina, San Diego, USA; www.illumina.com). DNA samples with GenCall scores <0.25 at any locus were considered “no calls”. A DNA sample was deemed to have failed if it generated genotypes at <95% of loci. A SNP was deemed to have failed if fewer than 95% of DNA samples generated a genotype at the locus. To ensure quality of genotyping, a series of duplicate samples were genotyped in the same batches. For all SNP assays >99% concordant results were obtained.

**LIST - Genes investigated**

ATP-binding-casette transporter C1 (ABCC1),

Apolipoprotein E (APO E),

Apurinic-Apyramidinic Endonuclease 1 (APEX1),

Ataxia telangiectasia mutated (ATM),

Ataxia telangiectasia and Rad3-related protein (ATR),

Brain derived neurotrophic factor (BDNF),

Catalase (CAT),

Cyclin-dependent kinase inhibitor 1A (CDKN1A),

Connective-tissue growth factor (CTGF),

D-amino-acid oxidase activator (DAOA),

Dopamine receptor D1 (DRD1),

Epidermal growth factor receptor (EGFR),

Endothelial nitric oxide synthase (eNOS),

Excision repair cross-completing (ERCC4),

Glutathione reductase (GSR),

HUS1 checkpoint clamp component (HUS1),

Insulin-like growth factor 1 (IGF 1),

Insulin-like growth factor 1 receptor (IGF1R),

Insulin-like growth factor 2 (IGF 2),

Interleukin 16 (IL16),

Insulin receptor substrate 1 (IRS1),

DNA ligase 4 (LIG 4),

Mitogen-activated protein kinase kinase kinase 17 (MAP3K17),

Microcephalin (MCPH1),

Mouse double minute 2 homolog (MDM 2),

Methylguanine-DNA methyltransferase (MGMT),

Superoxide dismutase (MnSOD+SOD2),

Myeloperoxidase (MPO),

Macrophage scavenger receptor 1(MSR1),

Nei-endonuclease 8-like 3 (NEIL3),

NLAM1,

Nitric oxide synthase (NOS1),

Neurotrophin 3 (NTF3),

Platelet-derived growth factor A (PDGFA),

DNA-polymerase ε-subunit (POLE),

**Peroxisome proliferator-activated receptor γ (PPARg)**,

DNA-dependent protein kinase (PRKDC),

RAD51,

Regulator of telomere elongation helicase 1 (RTEL1),

SLCC4,

Transforming growth factor β1 (TGFB1),

Tumour protein 53 (TP53),

Ubiquitin-conjugating enzyme E2B (UBE2B),

Vascular endothelial growth factor A (VEGF A),

Werner syndrome ATP-dependent helicase (WRN),

X-ray repair cross-complementing protein 1 (XRCC 1),

X-ray repair cross-complementing protein 3 (XRCC 3),

X-ray repair cross-complementing protein 5 (XRCC 5),

X-ray repair cross-complementing protein 6 (XRCC 6)

**Supplementary table 1** - results of the univariate and multivariate analyses

| *n=81* | *Cumulative incidence* | | | | *Univariate analysis* | | *Multivariate analysis Competing Risks Regression (CRR)* | | | |
| --- | --- | --- | --- | --- | --- | --- | --- | --- | --- | --- |
|  |  | | *36 m* | *60 m* | *P-value*  *(Fine Gray****)*** | ***Q-value***  ***(BH)*** | *HR (CI 95%)* | | *P* | |
|  |  |  |  |  |  |  | *CRR1* | *CRR2* | *CRR1* | *CRR1* |
| *Age* | *< 60 years (68/81)* | | *40%* | *46%* | ***0.0319*** |  |  |  |  |  |
|  | *>= 60 years (13/81)* | | *54%* | *54%* |  |  |  |  |  |  |
| *SNP*  *PPARG*  rs2120825 | *TT+TG (18/81)* | | *67%* | *73%* | ***0.0004*** | ***0.0868*** | ***2.81***  ***(1.24-6.37)*** | ***2.65 (1.34-5.23)*** | ***0.013*** | ***0.005*** |
|  | *GG (63/81)* | | *35%* | *40%* |  |  |  |  |  |  |
| *Diagnostic* | *RRIL (44/81)* | | *42%* | *48%* | */* | */* |  | |  | |
|  | *RICI (32/81)* | | *21%* | *36%* |  |  |  |  |  |  |
| *Smoking status* | *Smoking (12/38)* | | *67%* | *67%* | ***0.0205**** |  |  | |  | |
|  | *No smoking (26/38)* | | *38%* | *47%* |  |  |  |  |  |  |
| *Tumor location* | *Temporal (36/81)* | | *44%* | *47%* | *0.7159* |  |  | |  | |
|  | *Frontal (45/81)* | | *40%* | *48%* |  |  |  |  |  |  |
| *therapies* | *RT only (28/81)* | | *36%* | *40%* | *0.5680* |  |  | |  | |
|  | *Concomitant CT (17/81)* | | *47%* | *53%* |  |  |  |  |  |  |
|  | *CT after RT (36/81)* | | *44%* | *51%* |  |  |  |  |  |  |
| *Tumor molecular characteristics* | *1p19q* | *Yes (18/57)*  *No (39/57)* | *51%*  *41%* | *58%*  *47%* | *0.4556* |  |  | |  | |
|  | *IDH1* | *Yes (37/57)*  *No (20/57)* | *35%*  *55%* | *41%*  *60%* | *0.3874* |  |  | |  | |
| *Other cardiovascular risk factors* | *Arterial hypertension* | *Yes (11/68)*  *No (11/68)* | *45%*  *42%* | *45%*  *46%* | *0.5621* |  |  | |  | |
|  | *Diabetes* | *Yes (2/65)*  *No (63/65* | *40%*  *43%* | *40%*  *46%* | *0.8596* |  |  | |  | |
|  | *Hypercholesterolemia* | *Yes (10/68)*  *No (58/68)* | *30%*  *45%* | *30%*  *48%* | *0.8134* |  |  | |  | |

*** smoking status is not included in the competing risks regression model due to the large amount of missing data (43/81).


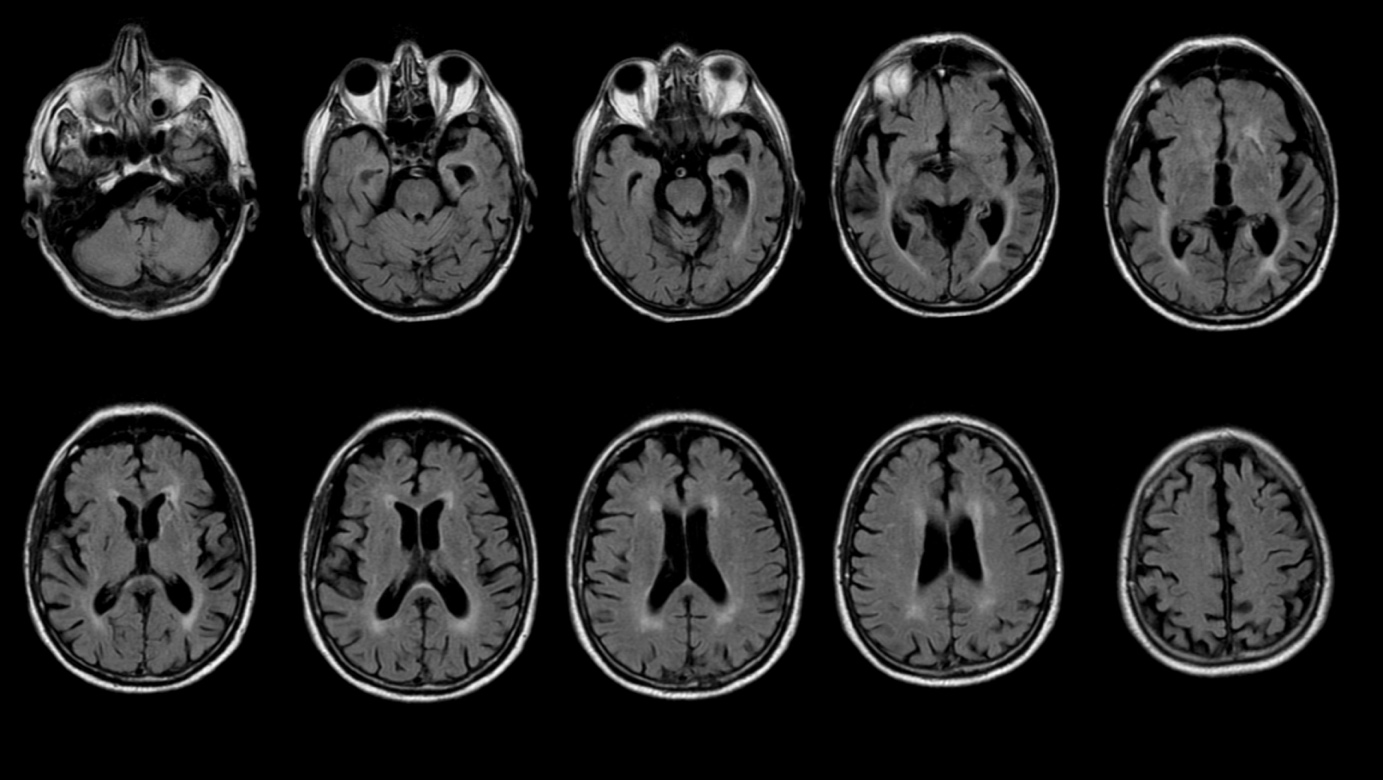


**Supplementary figure 1)** FLAIR series demonstrating mild degree of post-radiotherapy leukoencephalopathy; total of 8 points in the modified Scheltens rating scale

| **Modified Scheltens rating scale** | | |
| --- | --- | --- |
| Periventricular hyperintensities (5/6) | | |
| Caps occipital | 2 | 1: right or left hemisphere confluent lesion  2 : bilateral confluent lesion |
| Caps frontal | 2 |  |
| Band lateral ventricles | 1 |  |
| White matter hyperintensities (3/10) | | |
| frontal | 1 | 1: right or left hemisphere confluent lesion  2 : bilateral confluent lesion |
| occipital | 0 |  |
| temporal | 1 |  |
| insular | 1 |  |
| parietal | 0 |  |
| Basal ganglia hyperintensities (0/10) | | |
| caudate nucleus | 0 | 1: right or left hemisphere confluent lesion  2 : bilateral confluent lesion |
| putamen | 0 |  |
| globus pallidus | 0 |  |
| thalamus | 0 |  |
| internal capsule | 0 |  |
| Infra-tentorial foci of hyperintensities (0/4) | | |
| cerebellum | 0 | 1: right or left hemisphere confluent lesion  2 : bilateral confluent lesion |
| midbrain | 0 |  |


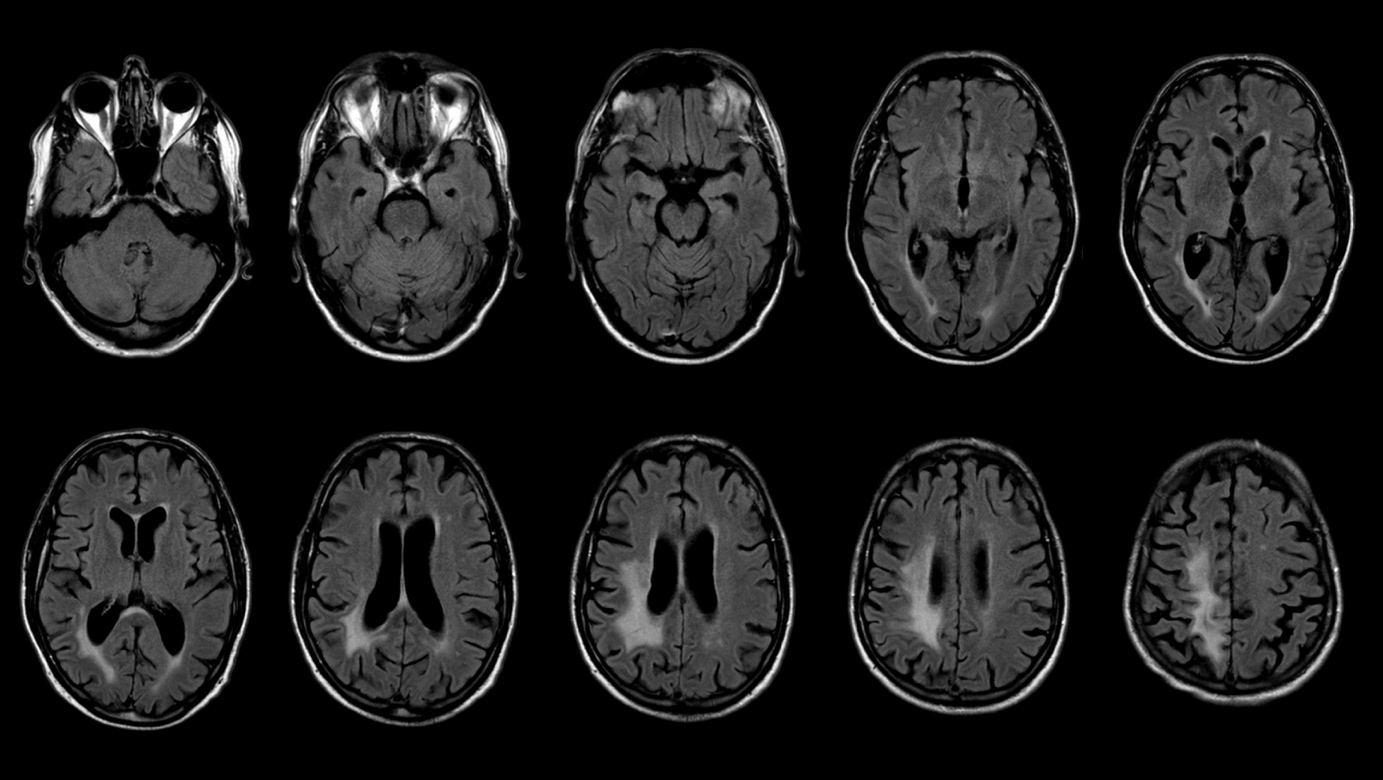


**Supplementary figure 2)** FLAIR series demonstrating mild degree of post-radiotherapy leukoencephalopathy; total of 10 points in the modified Scheltens rating scale

| **Modified Scheltens rating scale** | | |
| --- | --- | --- |
| Periventricular hyperintensities (4/6) | | |
| Caps occipital | 2 | 1: right or left hemisphere confluent lesion  2 : bilateral confluent lesion |
| Caps frontal | 1 |  |
| Band lateral ventricles | 1 |  |
| White matter hyperintensities (6/10) | | |
| frontal | 1 | 1: right or left hemisphere confluent lesion  2 : bilateral confluent lesion |
| occipital | 2 |  |
| temporal | 1 |  |
| insular | 0 |  |
| parietal | 2 |  |
| Basal ganglia hyperintensities (0/10) | | |
| caudate nucleus | 0 | 1: right or left hemisphere confluent lesion  2 : bilateral confluent lesion |
| putamen | 0 |  |
| globus pallidus | 0 |  |
| thalamus | 0 |  |
| internal capsule | 0 |  |
| Infra-tentorial foci of hyperintensities (0/4) | | |
| cerebellum | 0 | 1: right or left hemisphere confluent lesion  2 : bilateral confluent lesion |
| midbrain | 0 |  |


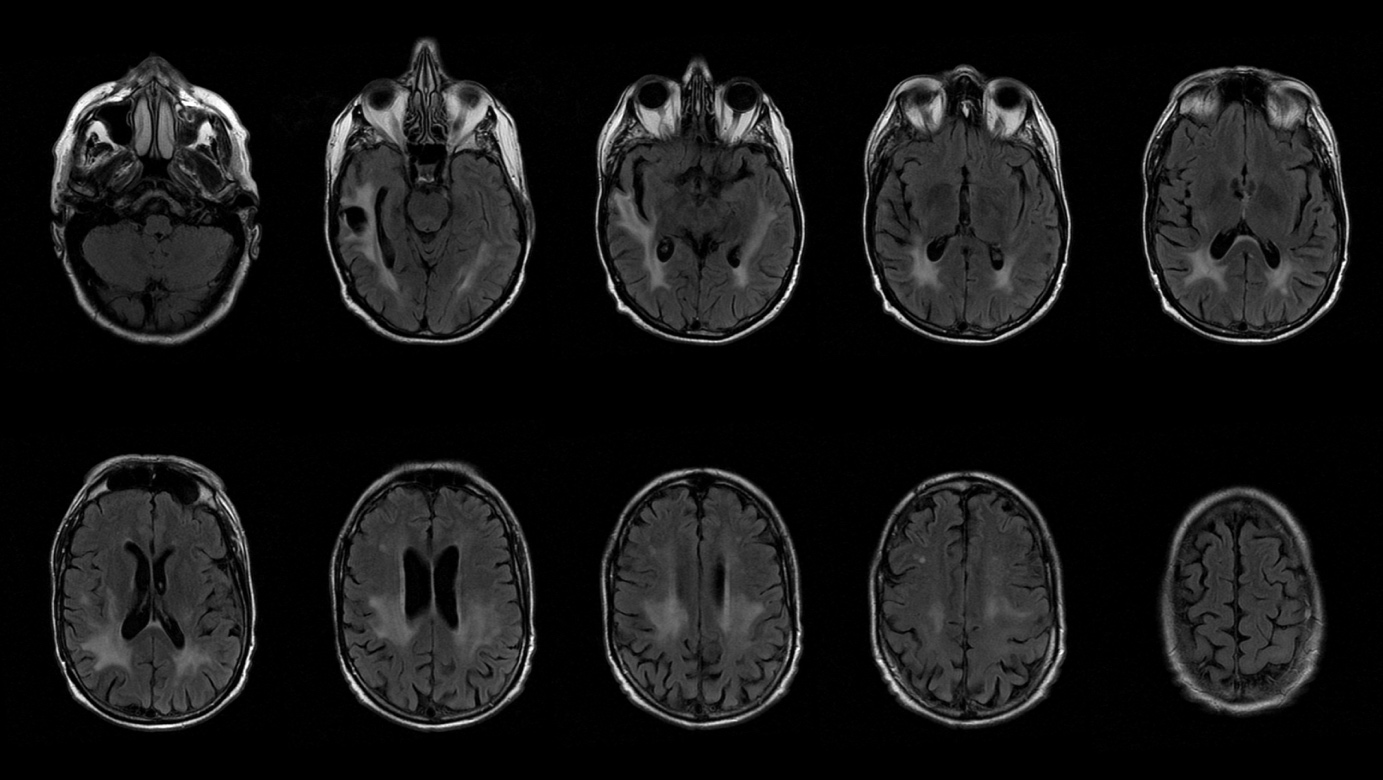


**Supplementary figure 3)** FLAIR series demonstrating mild degree of post-radiotherapy leukoencephalopathy; total of 15 points in the modified Scheltens rating scale

| **Modified Scheltens rating scale** | | |
| --- | --- | --- |
| Periventricular hyperintensities (6/6) | | |
| Caps occipital | 2 | 1: right or left hemisphere confluent lesion  2 : bilateral confluent lesion |
| Caps frontal | 2 |  |
| Band lateral ventricles | 2 |  |
| White matter hyperintensities (9/10) | | |
| frontal | 2 | 1: right or left hemisphere confluent lesion  2 : bilateral confluent lesion |
| occipital | 2 |  |
| temporal | 2 |  |
| insular | 1 |  |
| parietal | 2 |  |
| Basal ganglia hyperintensities (0/10) | | |
| caudate nucleus | 0 | 1: right or left hemisphere confluent lesion  2 : bilateral confluent lesion |
| putamen | 0 |  |
| globus pallidus | 0 |  |
| thalamus | 0 |  |
| internal capsule | 0 |  |
| Infra-tentorial foci of hyperintensities (0/4) | | |
| cerebellum | 0 | 1: right or left hemisphere confluent lesion  2 : bilateral confluent lesion |
| midbrain | 0 |  |
